# Histological Characteristics of Conjunctiva-Associated Lymphoid Tissue in Young and Adult Holstein Cattle

**DOI:** 10.3390/ani13223481

**Published:** 2023-11-11

**Authors:** Keigo Kosenda, Osamu Ichii, Yusuke Yamashita, Hiromichi Ohtsuka, Shigeo Fukuda, Yasuhiro Kon

**Affiliations:** 1 Laboratory of Farm Animal Pathophysiology, Department of Farm Animal Medicine, School of Veterinary Medicine, Rakuno Gakuen University, Ebetsu 069-0836, Japan; fukuda@rakuno.ac.jp; 2 Laboratory of Anatomy, Department of Basic Veterinary Science, Faculty of Veterinary Medicine, Hokkaido University, Sapporo 060-0818, Japan; ichi-o@vetmed.hokudai.ac.jp (O.I.); y-kon@vetmed.hokudai.ac.jp (Y.K.); 3 Laboratory of Agrobiomedical Science, Faculty of Agriculture, Hokkaido University, Sapporo 060-8589, Japan; 4 Nayoro Veterinary Clinical Center, Hokkaido Agricultural Mutual Aid Association, Nayoro 096-0072, Japan; jiji.shizuku@gmail.com; 5 Section of Large Animal Clinical Sciences, Department of Veterinary Medicine, Obihiro University of Agriculture and Veterinary Medicine, Obihiro 080-8555, Japan; ohtsuka@obihiro.ac.jp

**Keywords:** cow, conjunctiva, mucosa, lymphoid tissue, histology

## Abstract

**Simple Summary:**

Many animals exhibit immune cell aggregation in the conjunctiva which prevents the invasion of viruses and bacteria. However, the features and functions of conjunctival immune cell aggregates in cows are not fully understood. Microscopic observations confirmed conjunctival immune cell aggregation in calves and adult cows, similar to that in other animals. However, these structures of calves are small and poor in immune cells compared to those of adult cows. These results suggest that cows have a conjunctival local immune system similar to that of other animals, and that conjunctival immune function is weak in calves. These findings may help to prevent infectious diseases in cows.

**Abstract:**

The conjunctiva-associated lymphoid tissue (CALT) has been used as a target site for mucosal vaccinations in several animals. In this study, we compared the morphological features of CALT in the eyelid and third eyelid between Holstein calves and adult cows. In the eyelids, CALTs in the form of diffused lymphoid tissue (DLT) and lymphatic follicles (LF) were observed, where DLTs were dominant and LFs were scarce. The CALTs of cows comprised T-, B-cells, macrophages, and antigen-presenting cells (APCs). In particular, B-cells were dominant except in the eyelids of the calves. The epithelial layer covering the CALT is often discontinuous and lacks goblet cells. Cytokeratin18 is strongly expressed in the epithelial layer covering the CALT, except in the third eyelids of adult cows. IgA-positive cells were diffusely distributed in the lamina propria of the conjunctiva of the eyelids and third eyelids. The eyelid CALT area in calves was lower than that in adult cows. Furthermore, the CALT of calves had a lower cellularity of B-cells and a higher cellularity of macrophages than that of adult cows. These histological characteristics indicate that CALT plays a role in the mucosal immune-inductive and effector sites. Furthermore, lower cellularity of B-cells in the CALT of calves indicates that the function of CALT as a mucosal immune induction site is less developed in calves than in adult cows.

## 1. Introduction

Mucosa-associated lymphoid tissue (MALT) is an organized lymphoid tissue that is localized in the systemic mucosa of mammals [1]. The main role of MALT is the production of antigen-specific secretory IgA (SIgA), which prevents luminal microorganisms from penetrating mucous membranes [2]. Gut-associated lymphoid tissue (GALT), which includes Peyer’s patches, is the most common and representative MALT [3]. In several animals, nasopharynx-associated lymphoid tissue (NALT) [4], bronchus-associated lymphoid tissue (BALT) [5], and tonsils [6] are known as MALTs; however, species-specific differences in MALT localization remain unclear.

MALTs possess several unique morphological features for efficient mucosal antigen uptake and presentation. Briefly, the epithelial layer covering MALTs called follicle-associated epithelium (FAE), contains specific cells that are morphologically distinguished from normal epithelial cells, known as microfold cells (M cells) [7,8,9]. M cells possess short and scarce microvilli [10], and their basal membrane forms a pocket-like structure surrounding the immune cells [11]. By developing these morphological features, M cells efficiently absorb and transport antigens through mucosal membranes [12]. In addition, CD4^+^ or CD8^+^ T-, B-cells, macrophages, and antigen-presenting cells (APCs) are placed in MALTs to smooth the adaptive mucosal immune response [13]. MALT is morphologically classified into diffuse lymphoid tissue (DLT) and lymphatic follicles (LF). DLTs are diffusely arranged immune cells within the lamina propria. A recent study reported that genital organ-associated lymphoid tissues (GOALTs) composed of LFs or DLTs were found in the vaginal vestibules of Holstein cows and that B-cells were dominant in both LFs and DLTs [14].

The immunological functions of MALT have been used to control infectious diseases. Mucosal vaccination via antigen delivery to MALT cells has been widely used. In cows, intranasal vaccines against pathogens that cause respiratory diseases are already commercial [15,16]. Systemic vaccination can only induce IgG responses in the peripheral blood. In contrast, mucosal vaccination can induce an IgG response and antigen-specific IgA secretion in the mucous membrane. Mucosal vaccines can prevent the onset of mucosal infectious diseases because antigen-specific IgA traps infectious microorganisms before they enter the mucosal membrane [17]. Furthermore, MALTs located at different sites in the body are functionally involved with each other. B-cells activated in one mucosal organ move to different mucosal organs and secrete IgA [18]. This immunological connection between MALT cells is called the common mucosal immune system (CMIS) [2]. The effectiveness of vaccines inducing antigen-specific IgA responses at mucosal sites by inoculation into other mucosal sites has been studied in veterinary medicine. For example, intraocular immunization against bacteria that cause periodontitis increases salivary-specific IgA in dogs [19].

In humans and several other animals, eye-associated lymphoid tissues, including conjunctiva-associated lymphoid tissue (CALT) and lacrimal drainage-associated lymphoid tissue, have been identified by histological examinations [20,21,22]. CALT is thought to initiate the conjunctival and corneal immune response [23]. Eyedrop immunization can induce an immune response in mucosa far from the eye, and the intraocular administration of ovalbumin and cholera toxin results in an increase in antigen-specific IgA, not only in the eye mucosa but also in the nasal and vaginal mucosa in mice [24]. In addition, the conjunctiva is anatomically connected to the nasal mucosa via the lacrimal drainage system and lymphatics [25,26]. Thus, eye drops are expected to serve as a vaccine administration route for respiratory diseases in several animals. The inactivated eye drop influenza vaccine effectively and safely induces systemic IgG and the nasal mucosal immune response in mice [27]. In chickens, a Newcastle disease vaccine using eye drops has been generally used [28].

Infectious diseases, such as keratoconjunctivitis, pneumonia, diarrhea, and mastitis, are common causes of morbidity and mortality in dairy and beef cows. These diseases lead to the loss of milk and meat production due to reduced body gain and individual death; therefore, there has been a strong demand for prevention measures for infectious diseases using mucosal vaccines. In cows, GALT [29], NALT [30], BALT [31], larynx-associated lymphoid tissue [32], and GOALT [14] have been found. CALT has also been reported in cows [20]; however, its histological characteristics and potential as a mucosal immune-inductive and effector site have not been fully clarified. In addition, CALT expression was underdeveloped in young mice [23]. However, the presence and histological characteristics of CALT in young cows remain unclear. In this study, we histologically analyzed the CALT of cows and compared its morphological characteristics between calves and adult cows to investigate whether CALT plays a role of MALT.

## 2. Materials and Methods

### 2.1. Animals and Sample Preparation 

The Holstein breed was used in the present study. We collected the samples from three cows aged 10–13 years which were euthanized for another research purpose at Hokkaido University (Sapporo, Japan) and four cows aged 7–9 years slaughtered at a meat-processing plant (Nichiro Chikusan Nayoro factory, Nayoro, Japan) as adult cows for histological analysis (*n* = 7). These cows were non-pregnant and showed no abnormal clinical signs on general physical examination before euthanasia or slaughter. Three male and five freemartin pre-weaning calves aged 4–51 days were used (*n* = 8). Rakuno Gakuen University (Ebetsu, Japan) accepts various clinical cases of cows from neighboring dairy farms. From these clinical cases, we euthanized the calves because of the poor prognosis for clinical conditions other than infection. The calves showed no clinical signs of infection affecting their immune system before euthanasia. The calves were euthanized in the presence of an anesthesiologist in accordance with the regulations of the Institutional Animal Care and Use Committee of Rakuno Gakuen University. Adult cows were sedated with xylazine hydrochloride (3.0 mg/kg body weight) intramuscularly, followed by general anesthesia with pentobarbital sodium (500 mg/kg body weight) administered intravascularly. The cows were euthanized by administering a saturated potassium chloride solution of at least 0.1 mL/kg body weight. Then, the jugular vein was exsanguinated. Calves were sedated with xylazine hydrochloride (0.2 mg/kg body weight) through an intravascular route followed by general anesthesia with thiamylal sodium (9 mg/kg body weight) via the intravascular route. The calves were euthanized by intravenously administering a suxamethonium chloride hydrate (0.2 mg/kg body weight).

**Figure 1:**
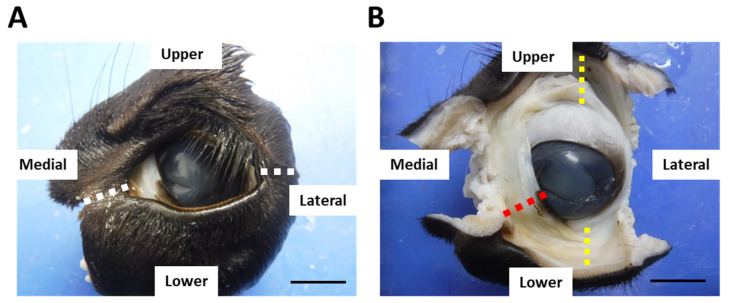
Conjunctival sample preparation. Left eyes from calf at 1 month of age. (**A**) One example of eye sample. White dotted lines: incision lines at the medial and lateral canthus. (**B**) Eye sample after medial and lateral canthus were cut. Upper and lower eyelids (eyelids) and the third eyelid were used for histological analyses. Yellow dotted lines: the upper and lower eyelids. Red dotted line: the third eyelid. Scale bars = 1 cm.

After euthanasia, the eyeballs with eyelids were obtained and fixed with 10% neutral-buffered formalin for >1 week. After fixation, the medial and lateral canthus were excised (Figure 1A). The upper, lower eyelids (eyelids), and third eyelids with conjunctiva were collected and used for histological analyses (Figure 1B). Trimmed tissues were dehydrated by series ethanol and embedded into paraffin. The animal experimentation procedures were approved by the Institutional Animal Care and Use Committee of the Faculty of Veterinary Medicine, Hokkaido University (approval no. 19-0097). The investigators adhered to the Guidelines for the Care and Use of Laboratory Animals of Hokkaido University, Faculty of Veterinary Medicine, and the Guideline for the Care and Use of Laboratory Animals of Rakuno Gakuen University.

### 2.2. Histological Examination

Paraffin sections (4 µm thick) were prepared and stained with hematoxylin and eosin (H&E) or periodic acid-Schiff (PAS). On histological observation under a light microscope, lymphoid clusters composed of >50 mononuclear cells in the conjunctival lamina propria were defined as CALT. 

Immunohistochemistry (IHC) was performed to detect immune cells, cytokeratin 18 (CK18) as an M cell marker, and bovine IgA. Detailed information on the antibodies, serum-blocking agents, and antigen retrieval methods used for IHC is shown in Table 1. Briefly, after deparaffinization and antigen retrieval, the sections were immersed in 0.3% hydrogen peroxide/methanol for 20 min and blocked with 10% normal goat serum (SABPO kit, Nichirei Bioscience; Tokyo, Japan) for 1 h at room temperature. Then, sections were incubated with each primary antibody at 4 ℃ overnight. After washing by phosphate buffered saline, the sections were incubated with secondary antibodies for 30 min at 20–25 °C. The sections were then treated with streptavidin-peroxidase (Nichirei Bioscience; Tokyo, Japan) for 30 min and 3,3′-diaminobenzidine tetrahydrochloride-hydrogen peroxide solution for 4 min. Finally, the sections were counterstained with hematoxylin.

### 2.3. Histoplanimetry

The appearance rate of the CALT was calculated as the number of CALT divided by the length of the observed conjunctiva in each section. The length of the observed conjunctiva and the area of the CALT were measured using an image analysis software (ImageJ 1.53, NIH, http://rsbweb.nih.gov/ij/ accessed on 19 August 2022). According to a previous report [8], each CALT was classified as LF or DLT without a follicular structure, and the appearance % was calculated. To measure the appearance rate of the CALT, more than 10 sections were observed. Measurement of the CALT area was performed for all observed CALTs, more than 20 in each group (calves eyelid: 28 CALTs, calves third eyelid: 44 CALTs, adult eyelid: 113 CALTs, adult third eyelid: 20 CALTs).

To assess the immune cell composition of the CALT, the total CALT area and the number of immunopositive cells for CD3, CD20, IBA1, and MHCⅡ in the CALT were counted, and the number of each immunopositive cells per 1000 μm^2^ were calculated. The rate of appearance of IgA-positive (^+^) cells was determined by dividing the number of IgA^+^ cells in the conjunctival lamina propria by the length of the conjunctiva observed in each section. The total CALT area, positive area for each immune cell marker, and length of the conjunctiva were measured using the ImageJ software (NIH). The sections containing relatively many CALTs were used for immune cell count to analyze at least 10 CALTs in each group. Although the same samples were used to count each immune cell, the number of CALTs analyzed were different between each immune cell depending on the condition of the sections (calves eyelid: 10–12 CALTs in 4 subjects, calves third eyelid: 11–19 CALTs in 3 subjects, adult eyelid: 19–27 CALTs in 3 subjects, adult third eyelid: 10–14 CALTs in 2 subjects). To measure the appearance rate of IgA^+^ cells, more than 10 conjunctival sections were examined.

### 2.4. Statistical Analysis

The results are expressed as the mean ± standard error. Each histological parameter was compared between calves and adult cows in the eyelids and third eyelids. LFs and DLTs appearance% were compared using Pearson’s chi-square test (*p* < 0.01), and other results were compared using the Mann–Whitney *U* test (*p* < 0.01).

## 3. Results

### 3.1. Histological Structure of CALT in Calves and Adult Cows

**Figure 2:**
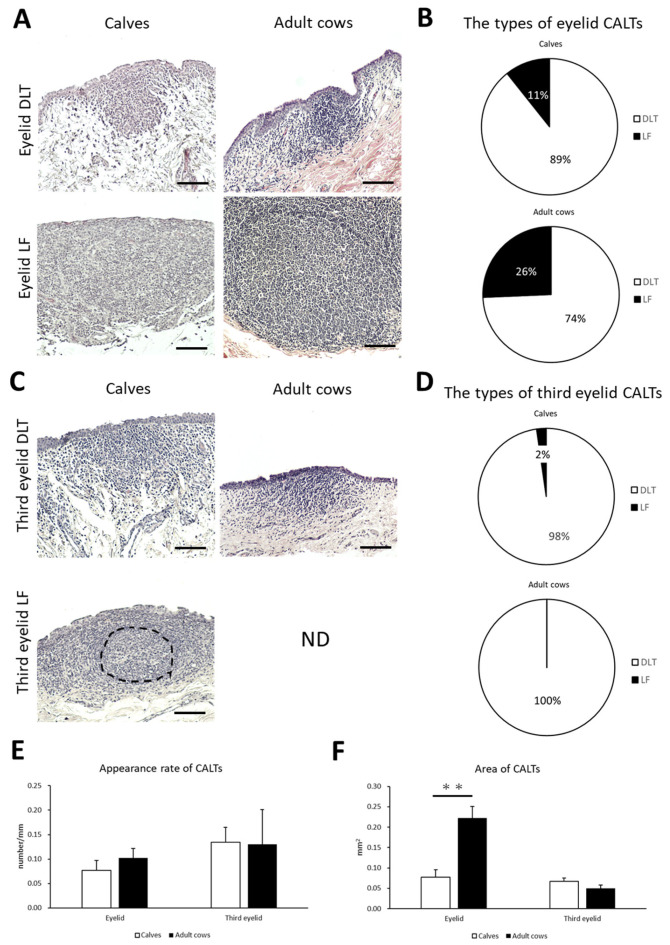
The types, the appearance rate, and the area of CALTs in calves and adult cows. (**A**) Diffuse lymphoid tissues (DLTs) and lymphatic follicles (LFs) in the eyelids of a calf (1 month of age) and adult cow (13 and 9 years of age, respectively). (**B**) The types of eyelid conjunctiva-associated lymphoid tissue (CALT). (**C**) DLTs and LFs in third eyelids of calf (3 weeks of age and 1 month of age, respectively) and adult cow (11 years of age). Hematoxylin and eosin (H&E) staining. ND: not detected. Dotted line indicates germinal center. Scale bars = 100 μm. (**D**) The types of third eyelid CALTs. (**E**,**F**) Comparison of the appearance rate or area of eyelid and third eyelid CALTs between calves and adult cows. *n* > 10 (panel (**E**)), 20 (panel (**F**)). Values = mean ± standard error (SE). **: significant difference between calves and adult cows, Mann–Whitney *U*-test (*p* < 0.01).

To clarify the histological characteristics of the CALT, the conjunctiva of the eyelid and third eyelid in preweaning calves and adult cows were observed. Lymphoid clusters were frequently observed on the eyelids of both calves and adult cows. They were localized in the conjunctival lamina propria in the form of DLT or LF (Figure 2A). DLTs indistinguishable from the surrounding tissue have a variety of shapes, such as flat, round, oval, trapezoidal, and rhomboidal. LFs are round- or oval-shaped lymphoid aggregates surrounded by connective tissue and rarely have germinal centers in both calves and adult cows. Most LFs were isolated, and aggregated LFs were rarely observed. Calves tended to have a lower LF than adult cows, but no significant differences were observed in the types of CALT between calves and adult cows (Figure 2B). 

On the third eyelids, DLTs were frequently observed in both calves and adult cows. However, LF was observed in only one case of a calf but not in adult cows (Figure 2C). Third eyelid DLTs also had flat, round, oval, trapezoidal, and rhomboidal shape, similar to eyelid DLTs. There was no significant difference in the types of CALT in the third eyelid between the calves and adult cows (Figure 2D). 

We then examined the appearance rate and area of CALTs in the eyelids and third eyelids to compare them between calves and adult cows. There was no significant difference in the appearance rate of CALTs between the calves and adult cows on either the eyelid or third eyelid (Figure 2E). There was no consistent trend in the distribution of CALT between the calves and adult cows. The eyelid CALTs area was significantly smaller in calves than in adult cows (*p* < 0.01). However, there was no significant difference in the area of the third eyelid CALTs between the calves and adult cows (Figure 2F). 

### 3.2. Immune Cell Composition of Eyelid CALT in Calves and Adult Cows

**Figure 3:**
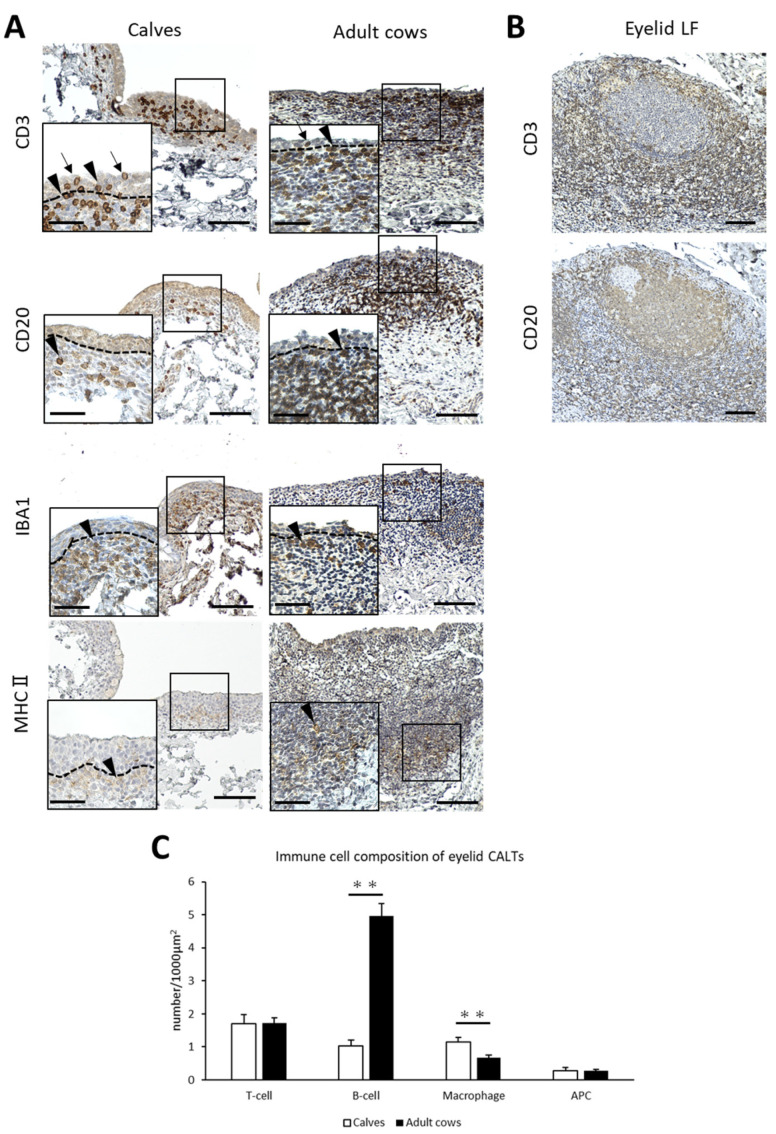
Immune cell composition of eyelid CALTs. (**A**) Immunohistochemistry for immune cell markers in eyelid CALTs in a calf (1 month of age) and adult cow (11 years of age). CD3: CD3^+^ T-cells, CD20: CD20^+^ B-cells, IBA1: IBA1^+^ macrophages, MHCⅡ: MHCⅡ^+^ APCs. Squares: magnified area. Black arrowheads: immunopositive cells. Black arrows: CD3^+^ T-cells observed between conjunctival epithelial cells. Dotted lines: border of epithelial layers and lamina propria. Scale bars = 100 and 50 μm (low and high magnifications, respectively). (**B**) IHC for CD3 and CD20 in eyelid LF of adult cow (9 years of age). Scale bar = 100 μm. (**C**) Comparison of the number of immunopositive cells for each immune cell marker in eyelid CALTs between calves and adult cows. *n* > 10. Values = mean ± SE. **: significant difference between calves and adult cows, Mann–Whitney U-test (*p* < 0.01).

To identify the immune cell types of the CALT in cows and the differences between calves and adult cows, IHC for immune cell markers was performed. CD3^+^ T-cells, CD20^+^ B-cells, and MHCⅡ^+^ APCs were diffusely distributed in the small eyelid DLTs of both calves and adult cows. However, IBA1^+^ macrophages tended to be distributed near the epithelial layer in the small DLTs (Figure 3A). In addition, T-cells were distributed not only in the lamina propria, but also between epithelial cells in both calves and adult cows (Figure 3A). In large DLTs and LFs, B-cells tended to congregate in the center of the CALTs and T-cells tended to be localized in the peripheral area (Figure 3B). Differences in immune cell distribution were not observed between calves and adult cows. 

The number of immunopositive cells for each immune cell marker per 1000 μm^2^ was calculated to compare the immune cell composition between the calves and adult cows. The number of B-cells in eyelid CALTs were significantly lower and the number of macrophages in eyelid CALTs were significantly higher in calves than in adult cows (*p* < 0.01; Figure 3C). 

### 3.3. Immune Cell Composition of Third Eyelid CALT in Calves and Adult Cows

**Figure 4:**
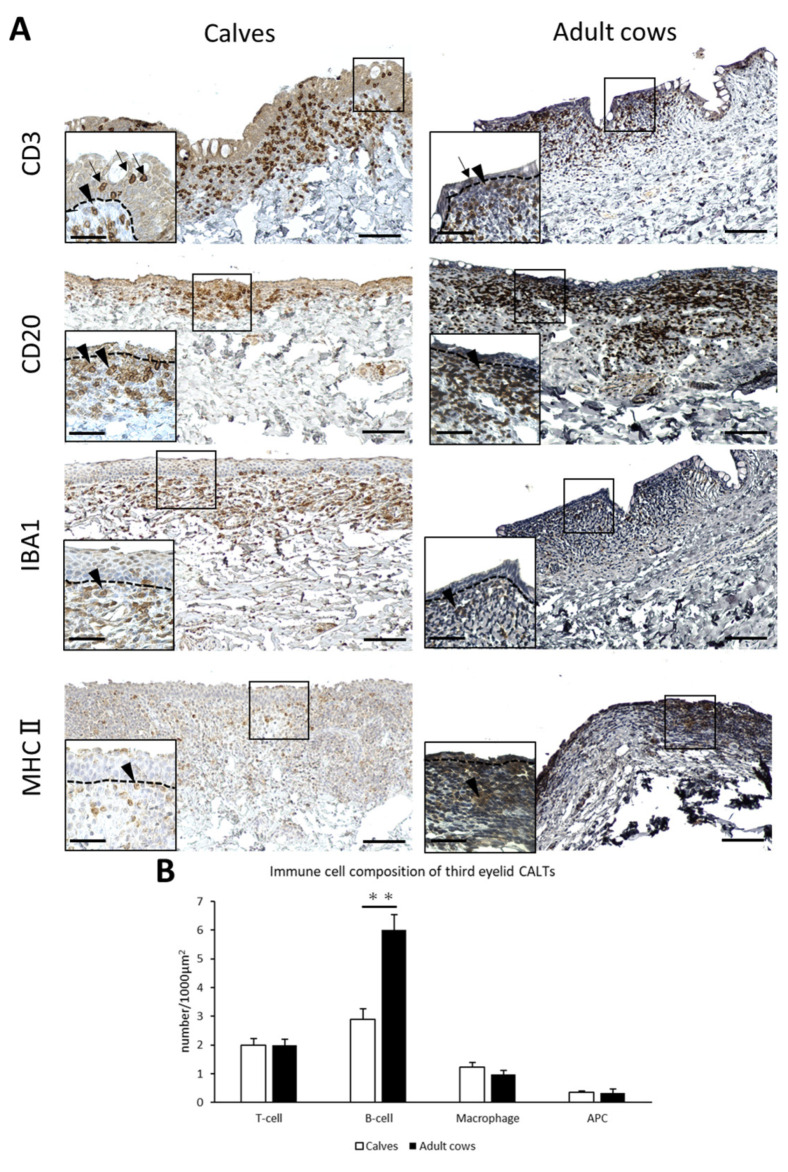
Immune cell composition of third eyelid CALTs. (**A**) IHC for immune cell markers in third eyelid CALTs in a calf (3 weeks of age) and adult cow (10 years of age). Squares: magnified area. Black arrowheads: immunopositive cells. Black arrows: CD3^+^ T-cells observed between conjunctival epithelial cells. Dotted lines: border of epithelial layers and lamina propria. Scale bars = 100 and 50 μm (low and high magnifications, respectively). (**B**) Comparison of the number of immunopositive cells for each immune cell marker in third eyelid CALTs between calves and adult cows. *n* > 10. Values = mean ± SE. **: significant difference between calves and adult cows, Mann–Whitney *U*-test (*p* < 0.01).

T-cells, B-cells, macrophages, and APCs were diffusely distributed in the DLTs of the third eyelid of both calves and adult cows (Figure 4A). Unlike the eyelids, there was no specific trend in T-, B-cells, and macrophages localization. T-cells were observed not only in the lamina propria, but also between epithelial cells in the third eyelid as well as in the eyelid. The cell distribution in the third eyelid LFs could not be investigated because they were scarce in the third eyelids. Differences in immune cell distribution were not observed between calves and adult cows. 

The number of B-cells in the third eyelid CALTs were significantly lower in calves than in adult cows (*p* < 0.01; Figure 4B).

### 3.4. Histological Characteristics and CK18 Expression of Epithelial Layer of CALT

**Figure 5:**
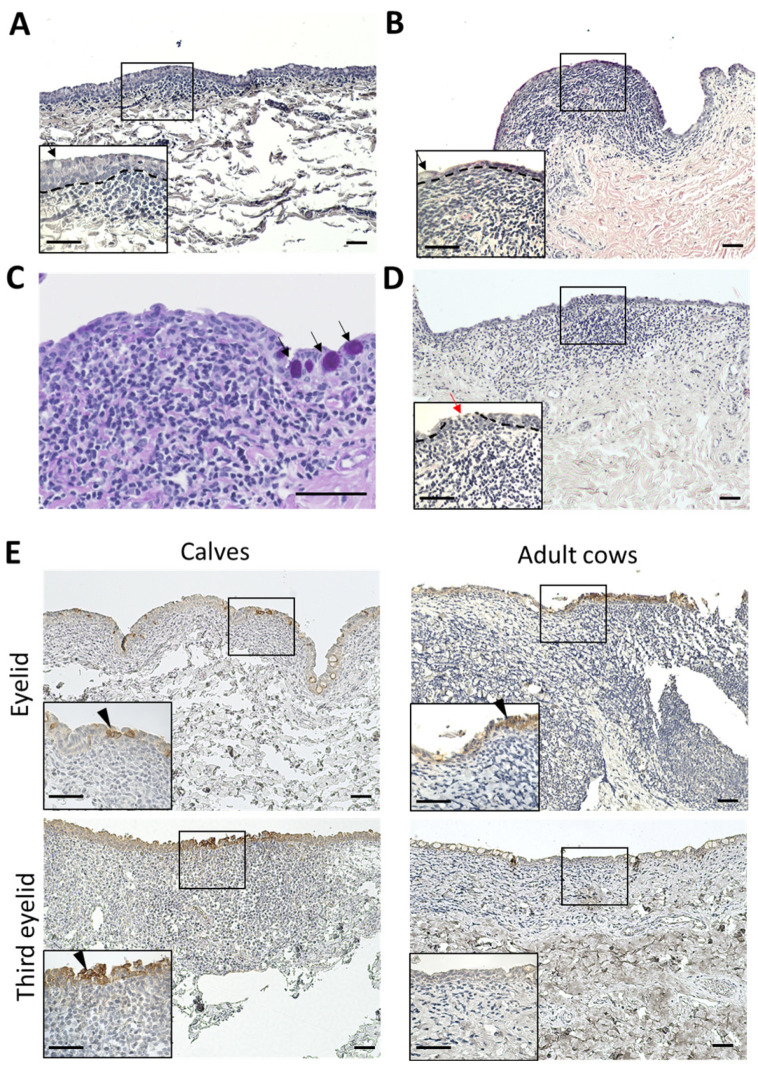
Histology and IHC for CK18 of epithelial layer covering CALTs. (**A**) Epithelial layer covering third eyelid CALT in a calf (2 weeks of age). (**B**) Epithelial layer covering third eyelid CALT in an adult cow (7 years of age). H&E staining. (**C**) Periodic acid-Schiff (PAS) staining of epithelial layer covering third eyelid CALT of a calf (1 month of age). Black arrows: goblet cells. (**D**) Epithelial layer covering eyelid CALT in an adult cow (11 years of age). Red arrows: cleavage of epithelial layer and infiltration of mononuclear cells. Dotted lines: border of epithelial layers and lamina propria. H&E staining. (**E**) IHC for CK18 in epithelial layer covering CALTs in a calf (eyelid: 1 week of age, third eyelid: 3 weeks of age) and adult cow (10 years of age). Arrowheads: Strong positive cells for CK18. Scale bars = 50 μm.

In the third eyelids, the epithelial layers of the calves were thicker than those of the adult cows (Figure 5A,B). Normal epithelial layers without CALT contain numerous goblet cells. However, the epithelial layer covering the CALTs lacked goblet cells in both the calves and adult cows (Figure 5A,B). Very few PAS-positive goblet cells were observed in the epithelial layers covering the third eyelid CALT (Figure 5C). All eyelid CALTs in the calves were covered with an epithelial layer. In some eyelid CALTs of adult cows, the epithelial layer covering the CALTs is thinned or cleaved. Mononuclear cells infiltrated into epithelial cleaves (Figure 5C). Some epithelial layers were dome- or mushroom-shaped and the lamina propria was filled with immune cells.

CK18 is an M cell marker of cows in Peyer’s patch [33]. To clarify the presence of M cells in the conjunctiva of cows, we observed the distribution of CK18^+^ cells in the conjunctiva using IHC. All conjunctival epithelia weakly expressed CK18. However, some conjunctival epithelial cells just above the CALT strongly expressed CK18 in the eyelids of calves and adult cows, and in the third eyelids of calves. These CK18 strong positive cells clustered in multiples rather than individually. In the third eyelids of adult cows, CK18 strong positive cells were not observed (Figure 5D). 

### 3.5. IgA^+^ Cells in Conjunctiva of Calves and Adult Cows

**Figure 6:**
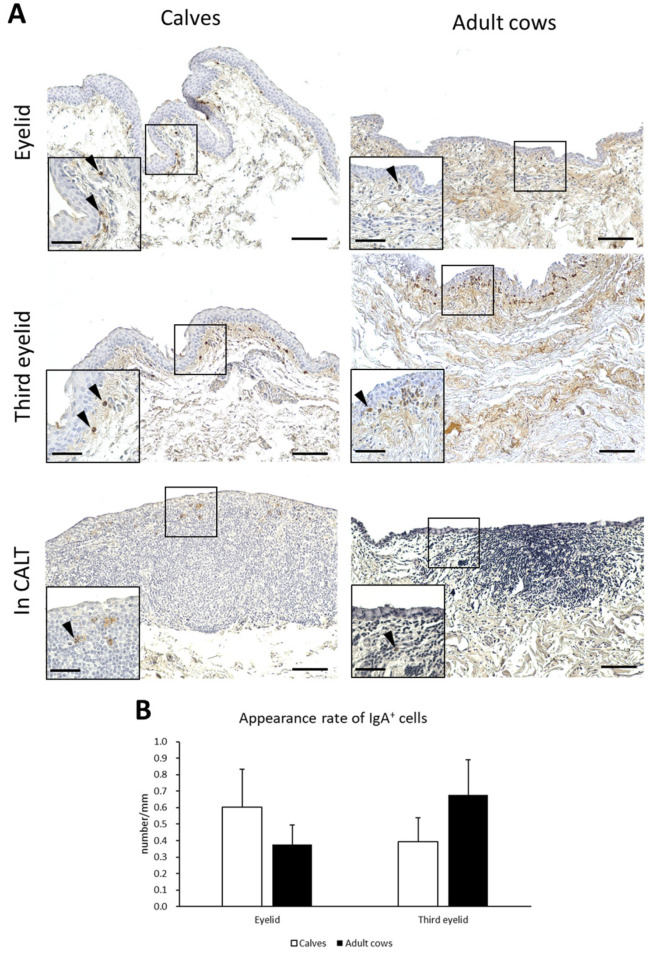
IgA^+^ cells in conjunctiva and its appearance rate. (**A**) IHC of conjunctiva for IgA in a calf (1 month of age) and adult cow (11 years of age). Arrowheads: IgA^+^ cells. Scale bars = 100 and 50 μm (low and high magnifications, respectively). (**B**) Comparison of the appearance rate of IgA^+^ cells between calves and adult cows. *n* > 10. Values = mean ± SE.

IgA production is a major function of mucosal immunity. To histologically verify IgA production in the conjunctiva of the cows, IHC for bovine IgA was performed. IgA^+^ cells having round, oval, and rarely spindle-shaped IgA^+^ cells were observed just below the conjunctival epithelium in the eyelids and third eyelids of both calves and adult cows. IgA^+^ cells were scattered throughout the conjunctival lamina propria and did not aggregate (Figure 6A). In contrast, IgA^+^ cells inside CALTs were very few and distributed in the peripheral area of the CALTs. The appearance rate of IgA^+^ cells in the eyelids of calves tended to be higher than in adult cows. In the third eyelids, the appearance rate of IgA^+^ cells in adult cows tended to be higher than that in calves. However, there was no significant difference between the calves and adult cows (Figure 6B).

## 4. Discussion

This study demonstrated the histological characteristics of CALT in cows and the differences between calves and adult cows. The presence of CALT in adult cows is already known, but its cell composition, FAE characteristics, IgA^+^ cell distribution, and presence in young cattle remained unclear. MALTs are functionally classified into immune inductive and effector sites. At inductive sites, B-cell proliferation and maturation occurs as a reaction to T-cell activation in the LF with a germinal center [13,34]. Thereafter, T- and B-cells move from the inductive sites to the effector sites and function as immune effector cells. Effector sites are distributed throughout the lamina propria in the form of DLT [35]. In this study, eyelid CALTs including LFs and DLTs were frequently observed in both calves and adult cows. Some LFs have germinal centers that are mainly composed of B-cells. In contrast, very few LFs were observed and most of the CALTs observed were DLTs in the third eyelids. Thus, we considered that the eyelids could function as mucosal immune-inductive sites in cows. However, the third eyelids of cows may not act as inductive sites, but mainly act as effector sites.

MALTs are primarily composed of T-cells, B-cells, macrophages, and APCs [13]. In the present study, these immune cells were observed in the eyelids and third eyelid CALTs of calves and adult cows. Furthermore, B-cells were abundant in the CALTs of adult cows. The main immune cells in the MALTs of cows are B-cells, such as GOALT [14], NALT [30], and GALT [29]. Thus, the immune cell composition of CALTs in cows is similar to that of other MALTs in cows already known. Furthermore, in some LFs, B-cells congregate at the germinal center. This result indicates the active proliferation of B-cells in CALT. Moreover, the ileum of calves has intraepithelial lymphocytes (IEL) mainly composed of T-cells [36] and ileal CD8^+^ intraepithelial T-cells increase in response to infection by *Cryptosporidium parvum* [37]. We showed that T-cells were distributed not only in the lamina propria of the CALT but also in the epithelial layer covering the CALT. This result shows that the conjunctival epithelium of cows has similar morphological features to the ileal mucosa at IELs although the functional difference remains to be determined. 

The normal mucosal epithelial layer contains many goblet cells that secrete mucin to prevent antigens from adhering to epithelial cells [38]. However, in many animals, FAE in the CALT lacks goblet cells to directly contact the tear film antigens in many animals [26]. In the present study, the conjunctival epithelial layer covering the third eyelid CALT, in both calves and adult cows, contained very few goblet cells, whereas the normal conjunctival epithelial layer in the third eyelids contained abundant goblet cells. The lack of goblet cells in cow CALTs is a feature similar to CALTs in other animals and facilitates the direct contact and uptake of antigens by epithelial cells in cow CALTs. Furthermore, in some adult cows, epithelial layers just above the eyelid CALTs are cleaved and occupied by immune cells. This discontinuous epithelial layer is similar to the CALTs of other animals [26,39], GOALT of cows [14], and NALT of cows [30]. The epithelial layer covering the CALTs of cows showed stronger CK18 expression than the normal epithelial layer, except for the eyelid CALTs of adult cows. CK18 is an intermediate filament protein known as a marker of M cells in Peyer’s patches [33]. Thus, the expression pattern of CK18 suggests that the CALTs of cows contain M-cell-like epithelial cells specialized in antigen uptake. However, the most reliable method for detecting M cells is ultrastructural observation [21]. The histological features of FAE in the CALT of cows indicate that they have characteristics that are different from the normal conjunctival epithelial layer. Further observations using electron microscopy are needed to clarify the presence of M cells and the fine features of antigen uptake in the CALT of cows.

In humans, IgA^+^ plasma cells are distributed throughout the conjunctival lamina propria [40]. In the present study, IgA^+^ cells were diffusely observed in the conjunctival lamina propria of cows, similar to that in humans. This suggests that the conjunctiva of cows produce local immunoglobulins. Generally, polymeric IgA (pIgA) produced by mucosal plasma cells combines with the poly-Ig-receptor (pIgR) distributed in the basolateral membranes of epithelial cells. IgA with pIgR is transported to the apical side via transcytosis. Finally, the IgA-pIgR complex is cleaved into pIgA by the extracellular fragment of pIgR termed the secretory component (SC) and is secreted onto the epithelial surface as SIgA [40]. The presence of pIgR and SC in the conjunctiva of cows has not yet been identified. Therefore, it is unclear whether IgA in the conjunctiva of cows is secreted into the conjunctival surface. In contrast, the CALTs of cows contain very few IgA^+^ cells. Therefore, we considered that immunoglobulin class switching to IgA might occur after B-cells leave CALTs aimed at mucosal immune effector sites in cows.

Generally, MALT cells are immature with scarce immune cells in neonatal individuals [13]. For example, the BALT levels in cows are absent in neonates and increase until 18 months of age in an age-dependent manner. In addition, the number of BALT in cows suffering from pneumonia was higher than that in normal healthy cows [31]. In this study, CALTs in adult cows had a higher area and number of B-cells than those in calves, although the number of CALTs did not differ between calves and adult cows. These results show that CALT has higher immune cellularity and that B-cell proliferation is more active in adult cows than in calves. Therefore, it is suggested that the function of CALT in calves as a mucosal immune inductive site is immature compared to that in adult cows. On the other hand, the number of macrophages in eyelid CALTs was lower in adult cows than in calves. This result suggests that innate immunity by macrophages is more active in the conjunctiva of calves than in adult cows. We cannot rule out the possibility that these results were influenced by sex differences because the calves used in this study were male or freemartin and the adult cows were female. However, all calves were preweaning before sexual maturation. Therefore, we believe that the differences in CALT development observed in this study were primarily due to age-related differences.

## 5. Conclusions

The CALT of cows have similar morphological characteristics to those of other already-known MALTs and CALTs of other animals. Additionally, the CALT is more developed in adult cows than in calves. These results suggest that the local mucosal immune responses in the conjunctiva and CALT play roles as mucosal immune inductive and effector sites in cows. In addition, the function of the CALT as a mucosal immune-inducing site is thought to be less developed in calves than in adult cows. We believe that it can be applied for the prevention of bovine Infectious diseases, such as eye drop vaccines targeting CALT. 

## Figures and Tables

**Table 1:** Antibodies and staining condition for IHC.

Antigen	Primary Antibody	Dilution	Blocking	Antigen Retrieval	Secondary Antibody
CD3	Rabbit monoclonal antibodies, ab16669 (Abcam)	1:800	10% NGS	TB 115 °C, 15 min	Goat anti-rabbit (SABPO kit, Nichirei)
CD20	Rabbit polyclonal antibodies, ab27093 (Abcam)	1:800	10% NGS	TB 115 °C, 15 min	Goat anti-rabbit (SABPO kit, Nichirei)
IBA1	Rabbit polyclonal antibodies, 013-27691 (Wako)	1:2400	10% NGS	CB 115 °C, 15 min	Goat anti-rabbit (SABPO kit, Nichirei)
MHCⅡ	Mouse polyclonal antibodies, BOV-CT2012 (Washington State University)	1:200	10% NGS	TB 115 °C, 15 min	Goat anti-mouse IgG (Abcam, ab6788) 1: 500(Biotinylated)
CK18	Mouse monoclonal antibodies, 65028 (Progen Biotechnik)	No need	10% NGS	TB 115 °C, 15 min	Goat anti-mouse IgG (Abcam, ab6788) 1: 500(Biotinylated)
IgA	Rabbit polyclonal antibodies, A10-108A (Bethyl Laboratories)	1:40,000	10% NGS	No need	Goat anti-rabbit (SABPO kit, Nichirei)

CB: citrate buffer pH6, TB: tris buffer pH9, NGS: normal goat serum.

## Data Availability

Data supporting the findings of this study are available from the corresponding author upon request.

## References

[1] Liebler-Tenorio E.M., Pabst R. (2006). MALT structure and function in farm animals. Vet. Res..

[2] Kiyono H., Fukuyama S. (2004). NALT-versus Peyer’s-patch-mediated mucosal immunity. Nat. Rev. Immunol..

[3] Forehielli M.L., Walker W.A. (2009). The role of gut-associated lymphoid tissues and mucosal defence. Br. J. Nutr..

[4] Spit B.J., Hendriksen E.G., Bruijntjes J.P., Kuper C.F. (1989). Nasal lymphoid tissue in the rat. Cell Tissue Res..

[5] Pabst R., Gehrke I. (1990). Is the bronchus-associated lymphoid tissue (BALT) an integral structure of the lung in normal mammals, including humans?. Am. J. Respir. Cell Mol. Biol..

[6] Belz G.T., Heath T.J. (1995). The epithelium of canine palatine tonsils. Anat. Embryol..

[7] Bockman D.E., Cooper M.D. (1973). Pinocytosis by epithelium associated with lymphoid follicles in the bursa of Fabricius, appendix and Peyer’s patches. An electron microscopic study. Am. J. Anat..

[8] Owen R.L., Bye W.A. (1984). The membranous (M) cell and the mucosal immune system. Ann. Rev. Med..

[9] Owen R.L., Jones A.L. (1974). Epithelial cell specialization within human Peyer's patches: An ultrastructural study of intestinal lymphoid follicles. Gastroenterology.

[10] Bye W.A., Allan C.H., Trier J.S. (1984). Structure, distribution, and origin of M cells in Peyer’s patches of mouse ileum. Gastroenterology.

[11] Neutra M.R. (1999). M cells in antigen sampling in mucosal tissues. Curr. Top. Microbiol. Immunol..

[12] Owen R.L. (1977). Sequential uptake of horseradish peroxidase by lymphoid follicle epithelium of Peyer’s patches in the normal unobstructed mouse intestine: An ultrastructural study. Gastroenterology.

[13] Cesta M.F. (2006). Normal structure, function, and histology of mucosa-associated lymphoid tissue. Toxicol. Pathol..

[14] Chuluunbaatar T., Ichii O., Masum M.A., Namba T., Islam M.R., Otani Y., Elewa Y.H.A., Kon Y. (2022). Genital organ-associated lymphoid tissues arranged in a ring in the mucosa of cow vaginal vestibules. Res. Vet. Sci..

[15] Osman R., Malmuthuge N., Gonzalez-Cano P., Griebel P. (2018). Development and function of the mucosal immune system in the upper respiratory tract of neonatal calves. Annu. Rev. Anim. Biosci..

[16] Rossi P.S., Mattei R.I., Schllemer N.R., Thomaz G.R., Antunes A.V., Virmond M.P., Taube M.J., Bertagnon H.G. (2021). The effect of bovine vaccines against respiratory viruses administered either intranasal or intramuscular on broncho-alveolar fluid cells of heifers. Vet. Q..

[17] Hill K.L., Hunsaker B.D., Towsend H.G., Littel-van den Hurk S.v.D., Griebel P.J. (2012). Mucosal immune response in newborn Holstein calves that had maternally derived antibodies and were vaccinated with an intranasal multivalent modified-live virus vaccine. J. Am. Vet. Med. Assoc..

[18] Xu-Amano J., Kiyono H., Jackson R.J., Staats H.F., Fujihashi K., Burrows P.D., Pillai S., McGhee J.R. (1993). Helper T cell subsets for immuno-globulin A responses: Oral immunization with tetanustoxoid and cholera toxin as adjuvant selectively induces Th2 cells in mucosa associated tissues. J. Exp. Med..

[19] Shimizu Y., Iwasaki T., Tajima T., Yuba E., Kono K., Watari S. (2017). Induction of antibody response in the oral cavity of dogs following intraocular (eye drop) immunization with *Porphyromonas gingivalis* cell lysate incorporated in pH-sensitive fusogenic polymer-modified liposomes. J. Vet. Med. Sci..

[20] Chodosh J., Nordquist R.E., Kennedy R.C. (1998). Comparative anatomy of mammalian conjunctival lymphoid tissue: A putative mucosal immune site. Dev. Comp. Immunol..

[21] Kageyama M., Nakatsuka K., Yamaguchi T., Owen R.L., Shimada T. (2006). Ocular defense mechanisms with special reference to the demonstration and functional morphology of the conjunctiva-associated lymphoid tissue in Japanese monkeys. Arch. Histol. Cytol..

[22] Knop E., Knop N. (2002). A functional unit for ocular surface immune defense formed by the lacrimal gland, conjunctiva and lacrimal gland drainage system. Adv. Exp. Med..

[23] Siebelmann S., Gehlsen U., Hüttmann G., Koop N., Bölke T., Gebert A., Stern M.E., Niederkorn J.Y., Steven P. (2013). Development, alteration and real time dynamics of conjunctiva-associated lymphoid tissue. PLoS ONE.

[24] Seo K.Y., Han S.J., Cha H.R., Seo S.U., Song J.H., Chung S.H., Kweon M.-N. (2010). Eye mucosa: An efficient vaccine delivery route for inducing protective immunity. J. Immunol..

[25] Liebich H.G., Sótonyi P., König H.E., König H.E., Liebich H.G. (2016). Eye (*organum visus*). Veterinary Anatomy of Domestic Mammals Textbook and Colour Atlas.

[26] Lohrberg M., Pabst R., Wilting J. (2018). Co-localization of lymphoid aggregates and lymphatic networks in nose- (NALT) and lacrimal duct-associated lymphoid tissue (LDALT) of mice. BMC Immunol..

[27] Kim E.D., Han S.J., Byun Y.H., Yoon S.C., Choi K.S., Seong B.L., Seo K.Y. (2015). Inactivated eyedrop influenza vaccine adjuvanted with poly(I:C) is safe and effective for inducing protective systemic and mucosal immunity. PLoS ONE.

[28] Hassanzadeh M., Abdoshah M., Yousefi A.R., Masoudi S. (2020). Comparison of the impact of different administration routes on the efficacy of a Thermoresistant Newcastle disease vaccine in chickens. Viral Immunol..

[29] Parsons K.R., Howard C.J., Jones B.V., Sopp P. (1989). Investigation of bovine gut associated lymphoid tissue (GALT) using monoclonal antibodies against bovine lymphocytes. Vet. Pathol..

[30] Meek H.C., Stenfeldt C., Arzt J. (2022). Morphological and phenotypic characteristics of the bovine nasopharyngeal mucosa and associated lymphoid tissue. J. Comp. Pathol..

[31] Anderson M.L., Moore P.F., Hyde D.M., Dungworth D.L. (1986). Bronchus associated lymphoid tissue in the lungs of cattle: Relationship to age. Res. Vet. Sci..

[32] Casteleyn C., Simoens P., Van den Broeck W. (2008). Larynx-associated lymphoid tissue (LALT) in young cattle. Vet. Immunol. Immunopathol..

[33] Hondo T., Kanaya T., Takakura I., Watanabe H., Takahashi Y., Nagasawa Y., Terada S., Ohwada S., Watanabe K., Kitazawa H. (2011). Cytokeratin 18 is a specific marker of bovine intestinal M cell. Am. J. Physiol. Gastrointest. Liver Physiol..

[34] Huang C. (2020). Germinal center reaction. Adv. Exp. Med. Biol..

[35] Yan Z., Wang J.B., Gong S.S., Huang X. (2003). Cell proliferation in the endolymphatic sac in situ after the rat Waldeyer ring equivalent immunostimulation. Laryngoscope.

[36] Wyatt C.R., Barrett W.J., Brackett E.J., Davis W.C., Besser T.E. (1999). Phenotypic comparison of ileal intraepithelial lymphocyte populations of suckling and weaned calves. Vet. Immunol. Immunopathol..

[37] Wyatt C.R., Brackett E.J., Perryman L.E., Rice-Ficht A.C., Brown W.C., O’Rourke K.I. (1997). Activation of intestinal intraepithelial T lymphocytes in calves infected with *Cryptospridium parvum*. Infect. Immun..

[38] Verdugo P. (1990). Goblet cells secretion and mucogenesis. Annu. Rev. Physiol..

[39] Crespo-Moral M., García-Posadas L., Lópes-García A., Diebold Y. (2020). Histological and immunohistochemical characterization of the porcine ocular surface. PLoS ONE.

[40] Knop E., Knop N., Claus P. (2008). Local production of secretory IgA in the eye-associated lymphoid tissue (EALT) of the normal human ocular surface. Invest. Ophthalmol. Vis. Sci..

